# CT-based deep learning radiomics signature for the preoperative prediction of the muscle-invasive status of bladder cancer

**DOI:** 10.3389/fonc.2022.1019749

**Published:** 2022-12-05

**Authors:** Weitian Chen, Mancheng Gong, Dongsheng Zhou, Lijie Zhang, Jie Kong, Feng Jiang, Shengxing Feng, Runqiang Yuan

**Affiliations:** ^1^ Department of Urology, Zhongshan People's Hospital, Zhongshan, China; ^2^ First Clinical Medical College, Guangdong Medical University, Zhanjiang, China

**Keywords:** radiomics, deep learning, bladder cancer, machine learning, convolutional neural network (CNN)

## Abstract

**Objectives:**

Although the preoperative assessment of whether a bladder cancer (BCa) indicates muscular invasion is crucial for adequate treatment, there currently exist some challenges involved in preoperative diagnosis of BCa with muscular invasion. The aim of this study was to construct deep learning radiomic signature (DLRS) for preoperative predicting the muscle invasion status of BCa.

**Methods:**

A retrospective review covering 173 patients revealed 43 with pathologically proven muscle-invasive bladder cancer (MIBC) and 130 with non–muscle–invasive bladder cancer (non- MIBC). A total of 129 patients were randomly assigned to the training cohort and 44 to the test cohort. The Pearson correlation coefficient combined with the least absolute shrinkage and selection operator (LASSO) was utilized to reduce radiomic redundancy. To decrease the dimension of deep learning features, Principal Component Analysis (PCA) was adopted. Six machine learning classifiers were finally constructed based on deep learning radiomics features, which were adopted to predict the muscle invasion status of bladder cancer. The area under the curve (AUC), accuracy, sensitivity and specificity were used to evaluate the performance of the model.

**Results:**

According to the comparison, DLRS-based models performed the best in predicting muscle violation status, with MLP (Train AUC: 0.973260 (95% CI 0.9488-0.9978) and Test AUC: 0.884298 (95% CI 0.7831-0.9855)) outperforming the other models. In the test cohort, the sensitivity, specificity and accuracy of the MLP model were 0.91 (95% CI 0.551-0.873), 0.78 (95% CI 0.594-0.863) and 0.58 (95% CI 0.729-0.827), respectively. DCA indicated that the MLP model showed better clinical utility than Radiomics-only model, which was demonstrated by the decision curve analysis.

**Conclusions:**

A deep radiomics model constructed with CT images can accurately predict the muscle invasion status of bladder cancer.

## Introduction

BCa is one of the world’s 10 most common cancers (1), of which more than 500,000 newly diagnosed cases and about 200,000 deaths are reported worldwide every year (2). Over 90% of all malignant cases of BCa are derived from urothelial carcinoma, which is recognized as the most prevalent histological type of BCa (3). BCa can be divided into two types depending on how deep the tumor has infiltrated: no-muscle-invasive and muscle-invasive. Depth of tumor infiltration affects the management and prognosis of patient, which means that an accurate preoperative staging is rather vital for making appropriate therapeutic decisions for patients with BCa (4, 5). The EAU guidelines recommend radical cystectomy for MIBC patients and bladder preservation for non- MIBC patients (6).

MIBC can be diagnosed by histopathology. A biopsy following transurethral resection of a bladder tumor (TURBT) and a cystoscopy serve as the gold standard for the diagnosis of BCa. However, cystoscopy can induce a variety of complications, such as urethral injury and urinary tract infection. Additionally, due to the limited positioning, the collected biopsy specimens are relatively less and fail to reflect the overall scope of the tumor, resulting in the underestimation of the stage of the tumor (7).

In addition, several studies have reported muscle infiltration during subsequent radical cystectomy in some of the patients diagnosed with stage T1 who underwent TURBT. As a result, those patients with inadequate staging may experience a recurrence of the disease due to inadequate treatment. Computed tomography (CT) often serves as a common non-invasive imaging modality to diagnose patients with suspected BCa. However, the accuracy of CT in preoperative staging only reaches 35-40%. MRI is more accurate than CT in this way, but its high cost, prolonged scanning times, and several utilization contraindications limit the widespread use of MRI. Therefore, the development of a more accurate technique to assess BCa invasiveness is required.

Radiomics is an emergent imaging analysis, in which the radiological images are quantified so as to provide novel imaging biomarkers (8). The radiomics can contribute to elevating the accuracy of diagnosis, prognosis, and prediction, especially in oncology (9).In recent years, DL approaches have made major breakthroughs in computer vision, benefitting from the rapid expansion of data volume and computing capacity. Among the deep learning models applied in image analysis, convolutional neural networks are widely adopted, through which effective feature data can be extracted from image data and the inner structure of feature data can be learned for classification. CNNs is capable of automatically learning deep features of the input data, involving attributes, contour characteristics, location information and other high-dimensional information of the image object, which greatly elevates the recognition rate of the object. For certain cancer diagnosis, deep learning algorithm have achieved expert-level performance in segmentation and classification of medical images (10, 11).Neural networks usually requires huge amounts of data for training, however, it is difficult to obtain the high-quality labeled medical image in the real world. Considering this, transfer learning acts as a useful strategy for employing CNNs to medical image categorization (12, 13). Multiple studies, for example, suggest that CNNs pretrained on ImageNet data may be utilized to develop medical image classification models (14, 15).We hypothesized that deep learning features extracted from CT images could be utilized to modify the radiomics models for predicting MIBC. Consequently, the purpose of this research is to investigate the potential of using DLR to develop ML models based on enhanced CT combined with radiomics for distinguishing MIBC from non- MIBC.

## Materials and methods

**Figure 1:**
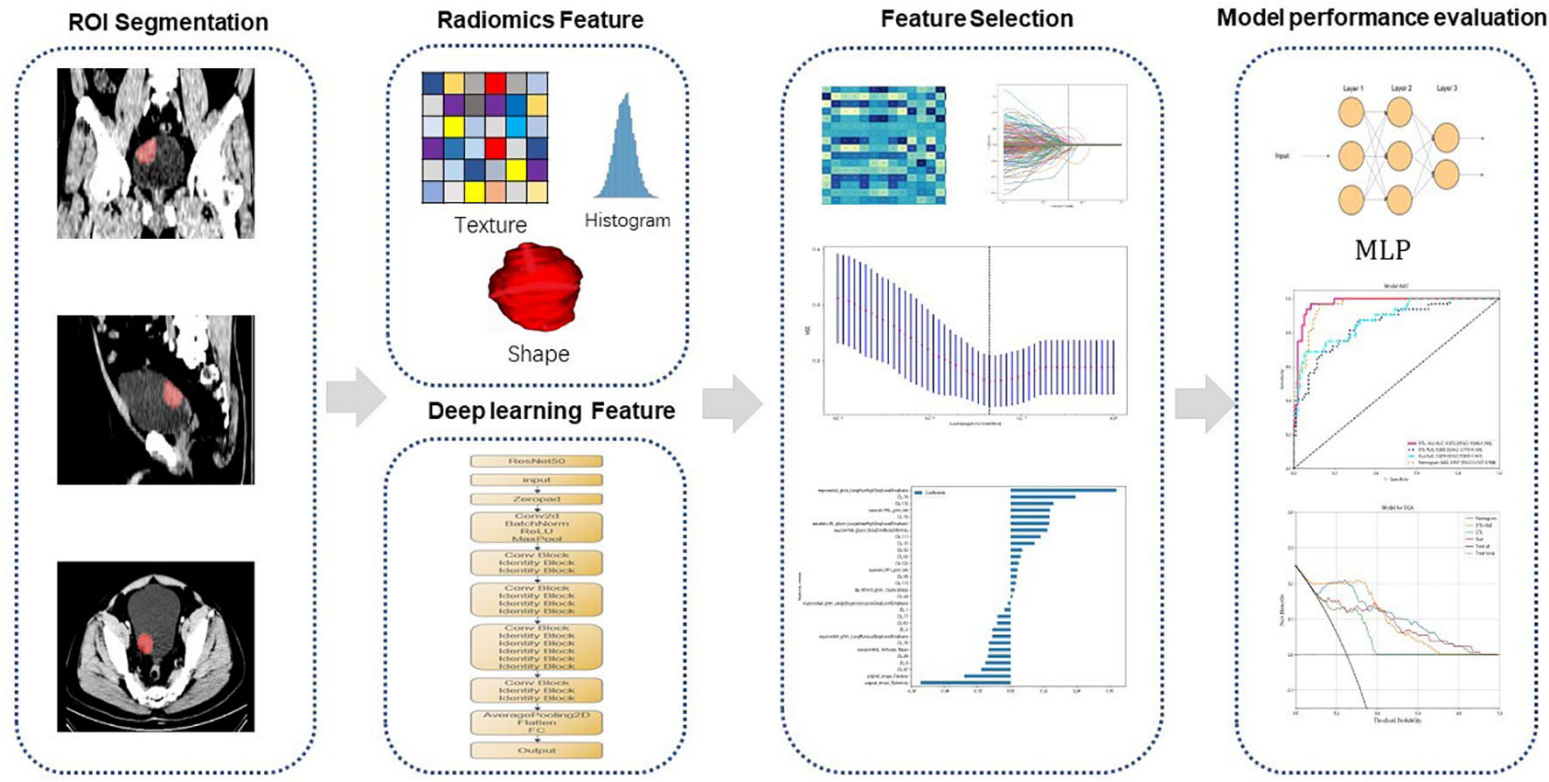
Workflow of our study. First, ROI segmentation is performed. Then, radiomics features and deep learning features are extracted and modeling and model test are performed. MLP Multilayer Perceptron.

**Figure 2:**
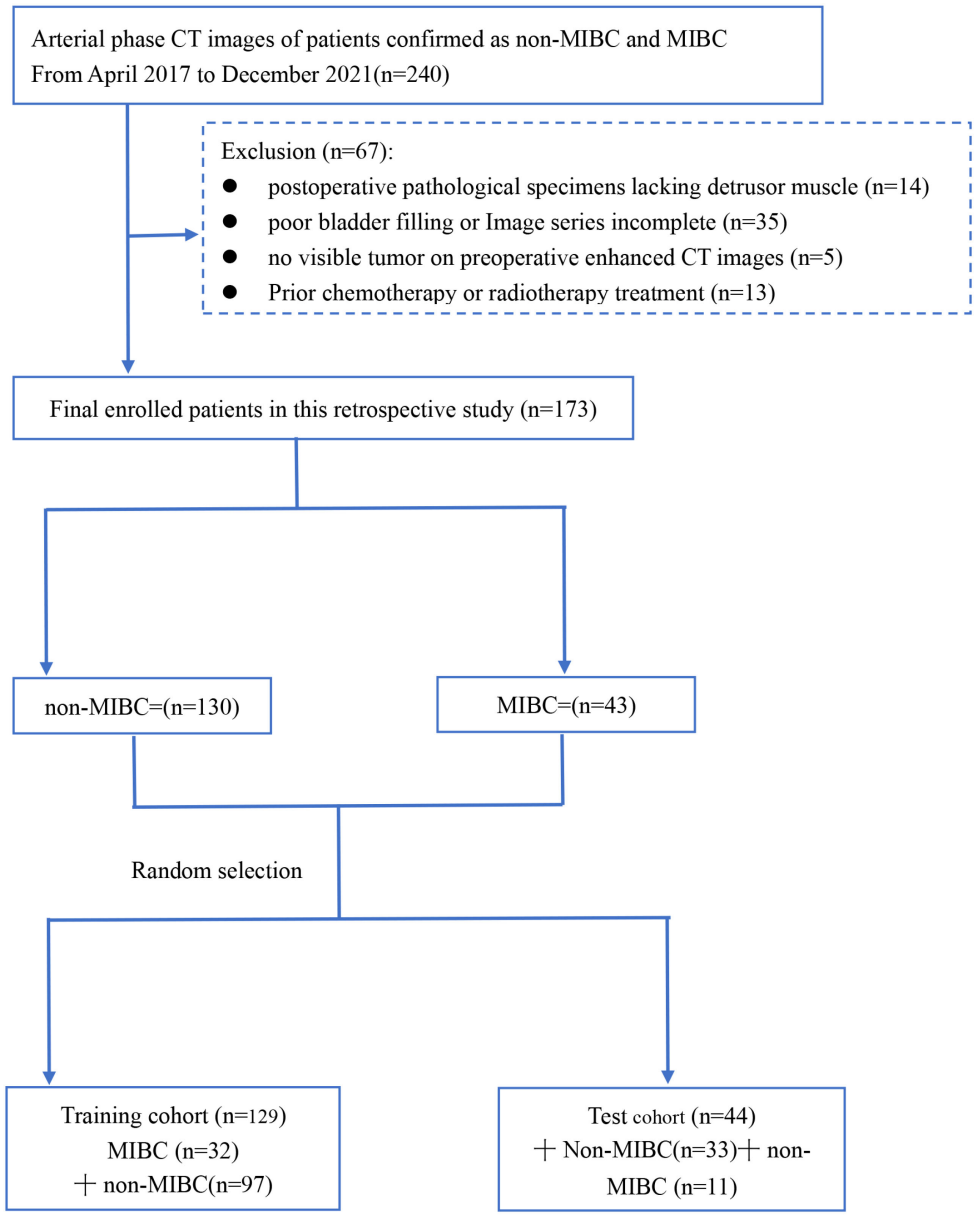
Flow diagram of patient enrollment and exclusion. muscle-invasive bladder cancer (MIBC), non–muscle–invasive bladder cancer (non- MIBC).

**Figure 1** shows our workflow. This retrospective analysis obtained ethical approval and waived the informed consent requirement. We retrospectively reviewed the medical charts of patients with BCa who received surgical treatments from April 2017 to December 2021. Inclusion criteria for patients were described as follows: (1) patients who underwent surgery and following treatment at Zhongshan people’s hospital from April 2017 to December 2021 with pathologically confirmed urothelial carcinoma; and (2) patients with histopathologically confirmed BCa by radical or partial cystectomy or TURBT within 4 weeks of CT scans. (1) patients had received chemotherapy or radiotherapy prior to surgery; (2) poor bladder filling or image quality; (3) tumors found during cystoscopy that were not visible on preoperative CT scans; and (4) postoperative pathological specimens lacking detrusor muscle. From the medical records, baseline clinical-pathologic data including age, sex, hydronephrosis status, and pathologic T stage were obtained from the medical records. The TNM staging system according to the 8th edition of the International Union Against Cancer. Patient enrollment and exclusion details are shown in **Figure 2**.

### Statistical analysis

In the training and testing cohorts, the clinical and pathological data were analyzed using Python 3.7.0 software. An analysis of diagnostic performance was conducted using ROC curves, area under the curve (AUC), and accuracy, sensitivity, and specificity as measures of diagnostic performance.

### Imaging equipment

CT imaging examinations were performed using the Philips ICT 256 and the Philips IQon spectral CT. Device parameters are as follows: tube voltage 120 kV or 130kv, with activated automatic tube current modulation, collimation 64 × 0.6 mm (Philips ICT 256) or 64 × 0.625 mm (Philips IQon spectral CT); pitch 0.9; image matrix 512× 512; slice thickness/slice interval 1 mm/1 mm (Philips ICT 256) or 0.683 mm/0.751 mm (Philips IQon spectral CT). Pixel spacing 0.605-0.751mm. The patients were scanned from the hemidiaphragm to the pelvic foor.The patients were injected with 100 mL of iopamidol or 80 mL of ioversol intravenously, followed by a 50 mL saline chaser at a rate of 3 mL/s. Corticomedullary-phase, nephrographic-phase, and excretory-phase images were obtained at 30 s, 60 s, and 300 s after the threshold was achieved in the thoracoabdominal aorta junction, respectively.

### Data preprocessing and tumor segmentation

Uneven voxel spacing in medical volumes is common due to the various acquisition protocols of each scanner, which we resolve using fixed resolution resampling algorithms. We manually delineated the region of interest for the entire tumor using ITK-SNAP (version 3.6.0; http://itk-snap.org). When a patient had multiple tumors, the maximum lesion was segmented for the features extraction. Lesions that could not be seen on the CT image or were less than 0.5 cm in size were not annotated.

### Interobserver reproducibility assessment

Interclass correlation coefficients (ICC) were used to measure ROI delineation reproducibility. A urologist manually delineated the region of interest (ROI) on CT images from 30 patients selected at random. Four weeks later, another urologist performed a repeat segmentation of the ROI region on these 30 patients. ICCs greater than 0.80 were considered to be good agreements.

## Results

### Patients’ characteristics

We collected 173 patients in this study. 129(75%) patients were used as the training cohort, and 44 patients were used as the test cohort. Training and test cohorts included the following clinical characteristics: age, number of lesions, therapeutic approach, number of lesions, and hydronephrosis status. We compared the clinical characteristics of the patients using an independent sample *t* test, Mann-Whitney *U* test, or *χ2* test, where appropriate. Clinical characteristics of patients in the training cohort and test cohort are shown in **Table 1**. **Table 2** shows the distribution of the clinical characteristics of the patients in the training and test sets. Differences were considered statistically Significant for p< 0.05. There were no significant differences of exact number of lesions, Gender, Therapeutic approach, Number of lesions and age in the training and test cohorts. As shown in **Table 2**, there were no differences between the training and testing cohorts in age, hydronephrosis status, gender, therapeutic approach, or number of lesions.

**Table 1:** Comparison of the clinical characteristics of the enrolled patients in the training and test sets.

Characteristics	Training cohort(n=129)				Test cohort (n=44)			
	MIBC	non- MIBC	ALL	P value	MIBC	non- MIBC	ALL	P value
Age	68.1250 ± 10.7065	66.7216 ± 12.9605	67.0698 ± 12.4142	0.58122896	62.2727 ± 8.7646	64.4545 ± 12.5202	63.9091 ± 11.6376	0.596155872
Exact number of lesions	1.2500 ± 0.6222	1.2577 ± 0.7675	1.2558 ± 0.7319	0.95891122	1.3636 ± 0.6742	1.1212 ± 0.3314	1.1818 ± 0.4458	0.119461671
Gender				0.91801102				0.100118555
Male	6(0.1875)	19(0.1959)	25(0.1938)		null	7(0.2121)	7(0.1591)	
Female	26(0.8125)	78(0.8041)	104(0.8062)		11(1.0000)	26(0.7879)	37(0.8409)	
Hydronephrosis				<0.001				<0.001
Yes	12(0.3750)	5(0.0515)	17(0.1318)		4(0.3636)	null	4(0.0909)	
No	20(0.6250)	92(0.9485)	112(0.8682)		7(0.6364)	33(1.0000)	40(0.9091)	
Therapeutic approach				0.05545129				0.014801282
Cystectomy	9(0.2812)	13(0.1340)	22(0.1705)		3(0.2727)	1(0.0303)	4(0.0909)	
TURBT	23(0.7188)	84(0.8660)	107(0.8295)		8(0.7273)	32(0.9697)	40(0.9091)	
Number of lesions				0.77081506				0.24399552
Multiple	6(0.1875)	16(0.1649)	22(0.1705)		3(0.2727)	4(0.1212)	7(0.1591)	
Single	26(0.8125)	81(0.8351)	107(0.8295)		8(0.7273)	29(0.8788)	37(0.8409)	

SD, standard deviation; MIBC, muscle-invasive bladder cancer; non- MIBC, non-muscle-invasive bladder cancer.

**Table 2:** Clinical characteristics of Bca patients in the training and test sets.

Characteristics	ALL	Training cohort (n=129)	Test cohort (n=44)	pvalue
Age, mean ± SD, years	66.2659 ± 12.2659	67.0698 ± 12.4142	63.9091 ± 11.6376	0.140423502
Exact number of lesions, mean ± SD, Number	1.2370 ± 0.6703	1.2558 ± 0.7319	1.1818 ± 0.4458	0.528753697
Gender				0.611104898
Male	32(0.1850)	25(0.1938)	7(0.1591)	
Female	141(0.8150)	104(0.8062)	37(0.8409)	
Hydronephrosis				0.476321023
Yes	21(0.1214)	17(0.1318)	4(0.0909)	
No	152(0.8786)	112(0.8682)	40(0.9091)	
Therapeutic apporach				0.204023849
Cystectomy	26(0.1503)	22(0.1705)	4(0.0909)	
TURBT	147(0.8497)	107(0.8295)	40(0.9091)	
Number of lesions				0.861599096
Multiple	29(0.1676)	22(0.1705)	7(0.1591)	
Single	144(0.8324)	107(0.8295)	37(0.8409)	

SD, standard deviation; MIBC, muscle-invasive bladder cancer; non- MIBC, non-muscle-invasive bladder cancer.

### Radiomics feature extraction

All CT images were resampled to a voxel size of 1 × 1 × 1 mm and discretized to grayscale with the bandwidth set to 5 before extracting radiomics features. The CT image extracts radiomic features using PyRadiomics (version:3.0.1). After PyRadiomics processing, we obtained a total of 107 categories of radiomics features. The 107 categories of radiomics features include 18 geometry features, 14 intensity features, and 75 texture features. Most of the features are based on the Imaging Biomarker Standardization Initiative’s Feature Definitions (IBSI) (16). 1735 radiomics features were obtained using LoG (σ:1.0, 2.0, 3.0), Wavelet, LBP3D, Exponential, Square, SquareRoot, Logarithm, and Gradient transform. The details of these parameters are shown at (https://pyradiomics.readthedocs.io/en/latest/customization.html).

### Feature selection

The intraclass correlation coefficients (ICCs) are used to evaluate the reproducibility of image features extracted from CT images. The features with ICCs > 0.80 were kept after analysis. In the end, 1616 radiomic features were retained after robustness assessment. After that, we calculated the correlation coefficient between each image feature using the Spearman rank correlation coefficient. One feature is randomly removed if the correlation coefficient between any two features is greater than 0.9. To preserve the capacity to depict features to the maximum degree possible, we use a greedy recursive deletion strategy for feature filtering, in which the feature with the greatest redundancy in the current cohort is removed each time.

### Deep learning feature extraction

**Figure 3:**
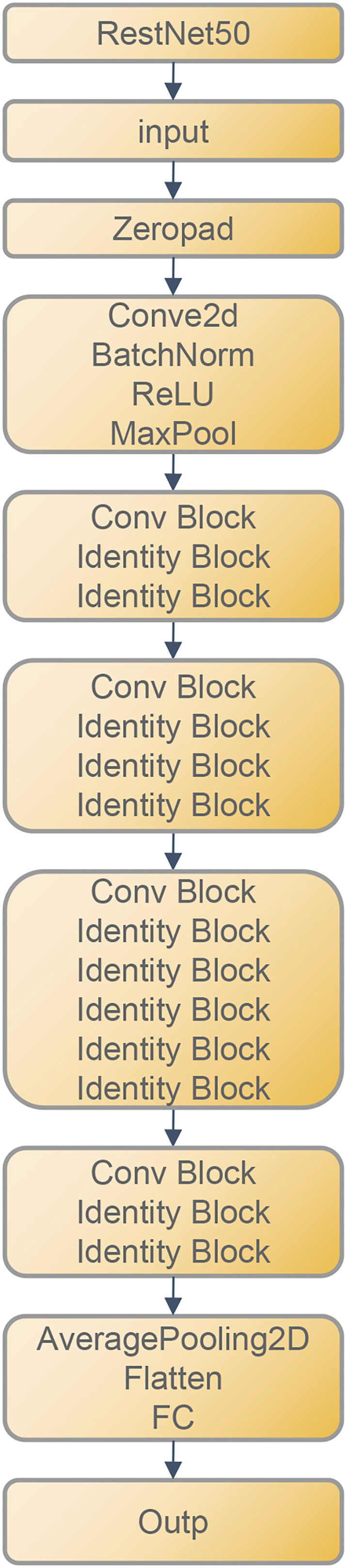
ResNet50 Architecture.

Before extracting the DTL features, images of the largest slice of the tumor area were selected to represent the patient. Then, the image grayscale values are normalized to the range (–1, 1) using a min-max transformation. The size of the cropped image was resized to 224*224 pixels. Due to the limited dataset, the resnet50 model (**Figures 3**) was trained on the ILSVRC-2012 dataset before extracting the deep learning features. Finally, the resnet50 model extracted and output 2048 features. In order to prevent overfitting and improve generalization, PCA was used to reduce the dimension of these features to 128. Pytorch is used to implement the deep learning framework of our network.

As a result of the leak of image data, we carefully cohort the learning rate to achieve better generalization. In this study, we adapted the cosine decay learning rate algorithm. Our learning rate is as follows:


$$\eta_{t}=\eta_{min}^{i}+\frac{1}{2}\left(\eta_{max}^{i}-\eta_{min}^{i}\right)\left(1+cos\left(\frac{T_{cur}}{T_{i}}\pi \right)\right)$$

$\eta_{min}^{i}=0$ , $\eta_{max}^{i}$ , =0.01, *T_i_* =30 Represents the minimum learning rate, the maximum learning rate, and the number of iteration epochs, respectively. Other hyperparameter configurations are as follows: optimizer: SGD, loss function: sigmoid cross entry.

### DLR signature building

**Figure 4:**
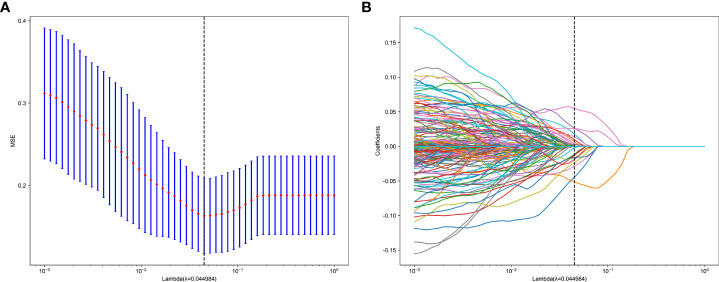
**(A)** MSE of cross test. **(B)** Based on the optimal λ value of 0.0449 with log(λ) = 0.044984 features were selected.

**Figure 5:**
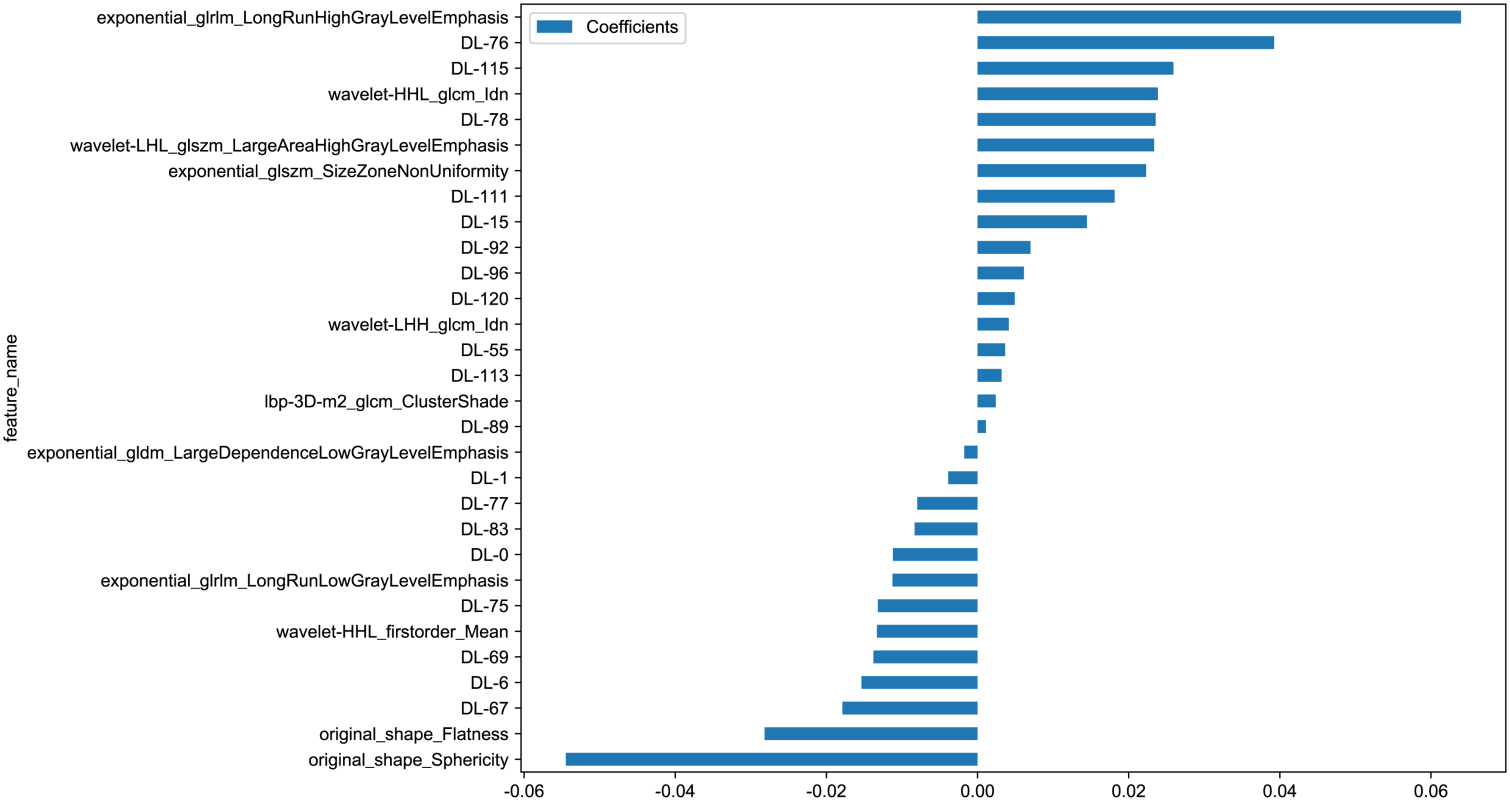
Rad-score histogram based on selected features.

We further explored whether model performance could be improved by fusing radiomics and deep learning features. Using 1735 radiomics features and 128 deep transfer learning features, we constructed a deep learning radiomics (DLR) signature. To obtain optimal features, we further reduce the dimensions of the fused features by LASSO after we have reduced and compressed the features using ICC, Spearman rank correlation coefficient, and PCA. In LASSO, the coefficients of many irrelevant features are set to zero entirely, depending on the weights λ. A minimum standard of 10-fold cross-validation was used in order to determine the optimal λ, where the final value of λ produced the smallest cross-validation error (**Figures 4A**, **B**). The DLR signature is constructed by linearly combining non-zero coefficients. By combining selected features weighted by their coefficients, Rad-scores is calculated by a linear combination of non-zero coefficients from selected features selected by LASSO. Ultimately, we selected 30 coefficients that contain 11 radiomics features and 19 deep learning features. **Figure 5** shows the Rad-score histogram.

DLR= 0.21000371276202448 -0.011196 * DL-0 -0.003878 * DL-1 -0.015362 * DL-6 +0.014477 * DL-15 +0.003652 * DL-55 -0.017882 * DL-67 -0.013772 * DL-69 -0.013185 * DL-75 +0.039252 * DL-76 -0.007971 * DL-77 +0.023570 * DL-78 -0.008322 * DL-83 +0.001110 * DL-89 +0.007005 * DL-92 +0.006138 * DL-96 +0.018146 * DL-111 +0.003174 * DL-113 +0.025918 * DL-115 +0.004912 * DL-120 -0.001756 * exponential_gldm_LargeDependenceLowGrayLevelEmphasis+0.063979*exponential_glrlm_LongRunHighGrayLevelEmphasis-0.011250*exponential_glrlm_LongRunLowGrayLevelEmphasis+0.022313*exponential_glszm_SizeZoneNonUniformity+0.002403*lbp-3D-m2_glcm_ClusterShade-0.028183*original_shape_Flatness-0.054490* original_shape_Sphericity-0.013320*wavelet-HHL_firstorder_Mean+0.023863*wavelet-HHL_glcm_Idn+0.004126*wavelet-LHH_glcm_Idn+0.023375*wavelet-LHL_glszm_LargeAreaHighGrayLevelEmphasis

### Model construction

Predictive models were constructed using radiomics combined with deep transfer transfer learning features. A variety of machine learning algorithms were tested, including LR、SVM、KNN、Random Forest、ExtraTrees、XGBoost、LightGBM, and deep learning-multilayer perception (MLP). With cross-test, all model parameters were optimized using scikit-learn’s GridSearch function. The MLP model uses three hidden layers with hidden dims of 256, 128, and 64. Adam optimizes and the learning rate is cohort to be 0.0001. Deep learning and radiomics signature building and model evaluation Described in detail in the **Supplementary Material**.

### Model performance evaluation

**Figure 6:**
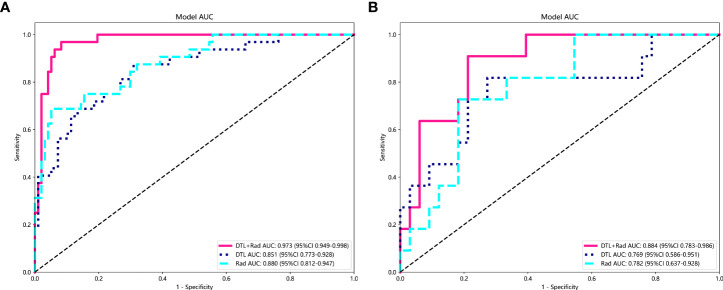
In the above figure, the performance of the MLP model is shown under the radiomics-only approach, the deep transfer learning approach, and the deep learning radiomics-based signature approach. Rad, Radiomics; DTL, Deep Transfer Learning; DLR, Deep Learning Radiomics. **(A)** Model performance in the training set. **(B)** Model performance in the test set.

**Figure 7:**
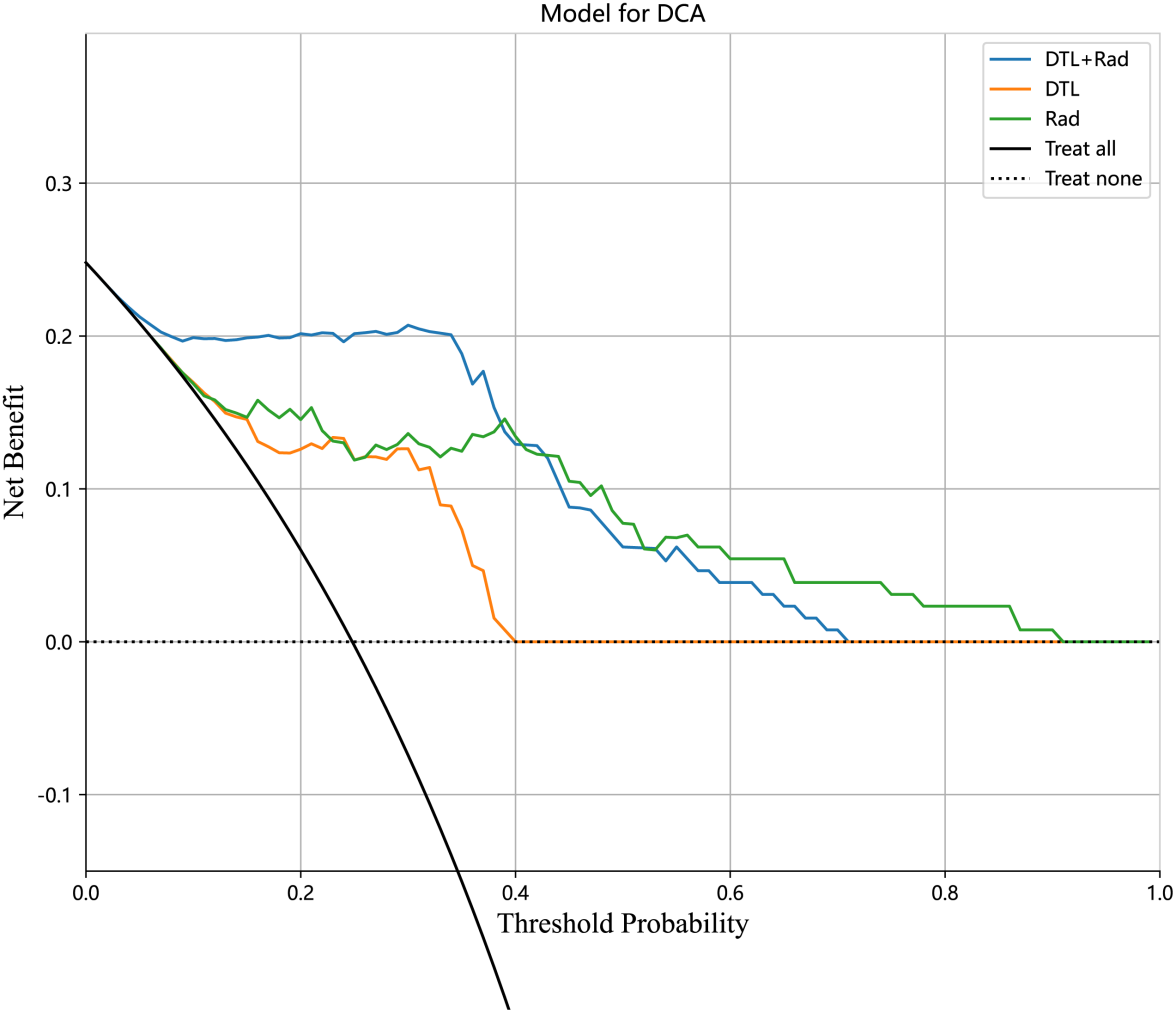
The decision curve analysis of all models for patients.

In the training and test cohorts, the MLP model performed well, achieving AUC of 0.973 (95% CI: 0.948-0.997) and 0.884 (95% CI: 0.783-0.985) respectively. In the training cohort, this MLP model had 93% accuracy, 96.87% sensitivity, 91.75% specificity, 79.48% NPV, and 98.88% PPV. In the test cohort, the model achieved accuracy, sensitivity, specificity, PPV, and NPV of 81.81%, 90.90%, 78.78%, 58.82%, and 96.29%, respectively. As shown in **Figures 6A, B**, ROC curves were constructed for each MLP model and the AUC was calculated for each ROC curve. The diagnostic performance of the above machine learning models is shown in **Table 3**. The results of the radiology-only approach and the deep transfer learning approach have been described in detail in the **Supplementary Material**. **Figure 7** shows the decision curve analysis based on, Radiomics-only Model, and the Deep Learning-Based Radiomics Model.

**Table 3:** Performance of the machine learning in the training and testing cohorts.

Model name	Accuracy	AUC	95% CI	Sensitivity	Specificity	PPV	NPV	Precision	Recall	F1 Score	Threshold	Data-cohort
SVM	0.992248	0.999356	0.9978 - 1.0000	1.000000	0.989691	0.969697	1.000000	0.969697	1.000000	0.984615	0.228051	train
SVM	0.727273	0.754821	0.5900 - 0.9196	0.727273	0.727273	0.470588	0.888889	0.470588	0.727273	0.571429	0.302342	test
KNN	0.775194	0.853254	0.7894 - 0.9171	0.812500	0.762887	0.530612	0.925000	0.530612	0.812500	0.641975	0.400000	train
KNN	0.772727	0.825069	0.7002 - 0.9500	0.636364	0.818182	0.538462	0.870968	0.538462	0.636364	0.583333	0.400000	test
RandomForest	0.976744	0.999517	0.9984 - 1.0000	1.000000	0.969072	0.914286	1.000000	0.914286	1.000000	0.955224	0.300000	train
RandomForest	0.613636	0.641873	0.4490 - 0.8348	0.636364	0.606061	0.350000	0.833333	0.350000	0.636364	0.451613	0.300000	test
ExtraTrees	1.000000	1.000000	nan - nan	1.000000	1.000000	1.000000	1.000000	1.000000	1.000000	1.000000	1.000000	train
ExtraTrees	0.704545	0.782369	0.6301 - 0.9347	0.727273	0.696970	0.444444	0.884615	0.444444	0.727273	0.551724	0.300000	test
XGBoost	0.945736	0.984214	0.9639 - 1.0000	0.937500	0.948454	0.857143	0.978723	0.857143	0.937500	0.895522	0.380025	train
XGBoost	0.704545	0.673554	0.4862 - 0.8609	0.636364	0.727273	0.437500	0.857143	0.437500	0.636364	0.518519	0.293565	test
LightGBM	0.968992	0.975515	0.9502 - 1.0000	0.937500	0.979381	0.937500	0.979381	0.937500	0.937500	0.937500	0.333712	train
LightGBM	0.772727	0.716253	0.5387 - 0.8938	0.545455	0.848485	0.545455	0.848485	0.545455	0.545455	0.545455	0.279661	test
MLP	0.930233	0.973260	0.9488 - 0.9978	0.968750	0.917526	0.794872	0.988889	0.794872	0.968750	0.873239	0.307806	train
MLP	0.818182	0.884298	0.7831 - 0.9855	0.909091	0.787879	0.588235	0.962963	0.588235	0.909091	0.714286	0.214518	test
LR	0.914729	0.975515	0.9521 - 0.9989	0.968750	0.896907	0.756098	0.988636	0.756098	0.968750	0.849315	0.276470	train
LR	0.727273	0.768595	0.6065 - 0.9306	0.727273	0.727273	0.470588	0.888889	0.470588	0.727273	0.571429	0.116137	test

AUC, area under the curve; CI, confidence interval; NPV, negative predictive value; PPV, positive predictive value.

## Discussion

In this study, deep learning and radiomic feature extraction from CT images were involved to develop MIBC and NO-MIBC classification models. An AUC of 0.78 (95% CI 0.637-0.928) was achieved by the MLP model constructed with radiomic features for assessing muscle invasion in BCa. The MLP model with radiomic features and deep learning features achieved better diagnostic performance, which was validated in the test cohort with an AUC value of 0.884 (95% CI 0.783-0.986).

The accurate staging of BCa is critical to minimizing the risk of under-or over-treatment. It is general for BCa patients to determine their stage by undergoing a CT scan. However, the diagnostic value of CT images for T-stage bladder cancer is limited due to the relatively poor soft tissue contrast of CT images. Usually, CT is used to evaluate bladder cancer at T3 and higher stages (5). The soft tissues are detected by MRI, as is more sensitive than a CT scan. Nevertheless, some downsides of MRI such as high cost and long exam duration, limit its widespread use for these applications. Most traditional medical image assessments are rooted in qualitative features like tumor density, enhancement pattern, regularity of tumor margins, and anatomical relationship to surrounding tissues. In contrast, radiomics analysis extracts high-throughput quantitative features from medical images, enabling the objective evaluation of medical images, which overcome the disadvantage of assessing medical images depending on radiologists’ experience. Radiomics as a promising method for preoperative staging of bladder cancer has displayed good results.

In the staging of bladder cancer, diagnostic potential has been demonstrated in radiomics. Kozikowski et al. (17), summarized eight relevant radiomics studies, showing high diagnostic performance of radiomics in predicting MIBC. There exhibited an overall 82% (95% CI: 77-86%) sensitivity and 81% (95% CI: 76-85%) specificity for predicting BCa muscle invasion status. Zhang et al. (18), collected 441 patients from two medical centers and randomly divided them into three cohorts: a training cohort of 293 patients; an internal test cohort of 73 patients; and an external test cohort of 75 patients. A logistic regression model was developed based on eight imaging radiomic features, of which the accuracy was validated on internal and external cohorts, achieving an AUC of 0.820 (95% CI 0.698-0.941) in the internal test cohort, and 0.784 (95% CI 0.674-0.893) in the external test cohort. Garapati et al. (19), developed a machine learning model for assessing bladder cancer staging by analyzing the morphological and textural characteristics of 83 bladder tumors. In both test cohorts, the AUCs of the ANN (Artificial Neural Network) classifier were 0.89 and 0.92, respectively. However, the above study only focused on the radiomic features of CT images.

Deep learning features of convolutional neural networks have been shown to be highly accurate in predicting survival, molecular subtypes, and recurrence in bladder cancer patients in many studies (20, 21). To date, fewer studies have examined the application of deep learning techniques in the preoperative staging of bladder cancer. Compared to radiomics, deep learning methods do not require manual outlining of ROI regions, which can reduce contour variations of different manual segmentations.

In order to develop a deep learning model to predict the muscle invasiveness of bladder cancer, Zhang et al. (22). used Enhanced CT Images. The model they studied obtained AUC of 0.791 (95% CI, 0.678-0.904) in an external validation cohort. Another study found that a CT-based deep learning convolution neural network model could achieve AUCs of 0.997 for preoperative prediction of BCa muscle invasive status (23). However, they only used deep learning features to assess the preoperative muscle infiltration status of Bca and did not incorporate radiomic features.Numerous studies have been done to combine radiomics with deep learning features to increase model prediction performance, despite the lack of interpretability of deep deep learning features.In this study, we propose a deep learning radiomics model for the accurate assessment of muscle invasion status in BCa patients preoperatively.The significance of the association between the radiomic and depth-learning features and the expression of the depth of tumor cell infiltration was quantified along with these features. The DLRS-based models attained an AUC of 0.884298(95% CI 0.7831-0.9855), which was the highest. To our knowledge, this is the first study to use deep learning radiomics to forecast the status of muscle infiltration in bladder cancer prior to surgery. Our study shows that the deep learning features extracted using resnet50 are complementary to the radiomics features.

The dimensions of raw features of the image extracted using resnet50 will reach 2048, posing great difficulty in the processing of the classifier in the later stage. PCA (Principal Component Analysis), also known as the principal component analysis method, is one of the most widely adopted algorithms for data dimensionality reduction. Since PCA can reduce the dimensionality of raw features while minimizing the loss of raw data,it was used in this study to reduce the dimensionality of deep learning features. We found that the discriminatory capability of the model incorporating deep learning features with radiomics features was enhanced in comparison to the model with radiomics only, demonstrating the value of deep learning features in the diagnosis of cancer. This result has also been found in other studies in the field of medical imaging (15, 24).

Convolutional neural networks as a deep learning technique are widely used for image recognition, which can automatically extract features from images based on convolutional operations, enabling them to detect subtle differences between MIBC and NO-MIBC. We selected Resnet50 as our deep learning feature extractor. With the residual block, Resnet network architecture is deeper and more capable of capturing subtle features in images in comparison to other CNN architectures. Therefore, Resnet is often employed for deep learning feature extraction from medical images, accompanied with excellent performance in this regard, which was also demonstrated by other medical image studies. The muscle invasion status of bladder cancer patients was predicted using deep learning features that were extracted through transfer learning techniques, with an AUC of 0.76 achieved in the test cohort. For the failure of the transfer learning model to outperform the radiomics-only model, a possible explanation is that the neural network was initialized with pre-trained model weights obtained from ImageNet rather than medical images. Despite the unsatisfactory results, this study provides evidence that deep convolutional neural networks can extract information used for BCa staging from CT images, laying the groundwork for future research in this field.

There were several limitations to this study. First, the present study is a retrospective analysis involving only one center; therefore, prospective studies from multiple centers will be necessary to validate the model’s predictive capabilities. Second, compared to other studies, our machine learning models constructed using only radiomics features performed poorly. The most likely explanation is that the imbalance between non- MIBC and MIBC in our sample affected the results. Third, we used only the arterial phase of the CT images in our analysis but we will add venous and excretory phase images in the future. Fourth, the model may be limited to some extent by a selection bias. Patients who received preoperative treatment and those with no visible tumor on preoperative enhanced CT images were excluded. As a result, it is unclear whether the proposed model will be effective for them. Due to the limitations of the data cohort, we chose a transfer learning strategy to train the resnet50 model, and in future studies we hope to avoid overfitting by pre-training the deep learning model on large medical images. Manual segmentation achieved good agreement in this study, and semantic segmentation models could be used in future studies to increase the reproducibility of the work (25).

In summary, by extracting radiomics features and DLR from CT images, we constructed various machine learning classifiers to distinguish MIBC from non- MIBC, and the results indicated that DLR Signature-based MLP provided great clinical utility in distinguishing non- MIBC from MIBC.

## Conclusions

We developed and validated deep learning and radiomics models based on CT images to distinguish MIBC from non-MIBC and found that they were superior to models constructed using radiomics features only.

## Data availability statement

The datasets presented in this article are not available for public access due to patient privacy concerns but can be obtained from the corresponding author on reasonable request approved by the institutional review boards of all participating institutions. Requests to access the datasets should be directed to RQY, zsrm2022@163.com.

## Ethics statement

The requirement for informed consent was waived, and this retrospective study was approved by the Zhongshan City People's Hospital Clinical Research and Animal Experiment Ethic Committee.

## Author contributions

Working concept or design: WTC, RQY. Data collection: DSZ, JK, FJ. SXF. Drafting the paper: WTC, MCG, WTC analysised the data. Making significant revisions to the paper:RQY, WTC. All authors contributed to the article and approved the submitted version.

## Acknowledgments

Our experiments were carried out on OnekeyAI platform. Thank OnekeyAI and it’s developers’ help in this scientific research work.

## Conflict of interest

The authors declare that the research was conducted in the absence of any commercial or financial relationships that could be construed as a potential conflict of interest.

## Publisher’s note

All claims expressed in this article are solely those of the authors and do not necessarily represent those of their affiliated organizations, or those of the publisher, the editors and the reviewers. Any product that may be evaluated in this article, or claim that may be made by its manufacturer, is not guaranteed or endorsed by the publisher.
