# Supplementary material for: CT-based deep learning radiomics signature for the preoperative prediction of the muscle-invasive status of bladder cancer
Source: Front Oncol. 2022 Dec 5;12:1019749. doi: 10.3389/fonc.2022.1019749 (PMC9761839; doi:10.3389/fonc.2022.1019749)
Supplement: Supplementary file 1 [file DataSheet_1.docx]

**Radiomics Signature**

After performing a Lasso feature screening, we input the final features for building risk models into machine learning models such as lr, svm, random forest, XGBoost, and others. To obtain the final Rad Signature in this instance, we use 5 fold cross verification.

**Radiomics model valuation**


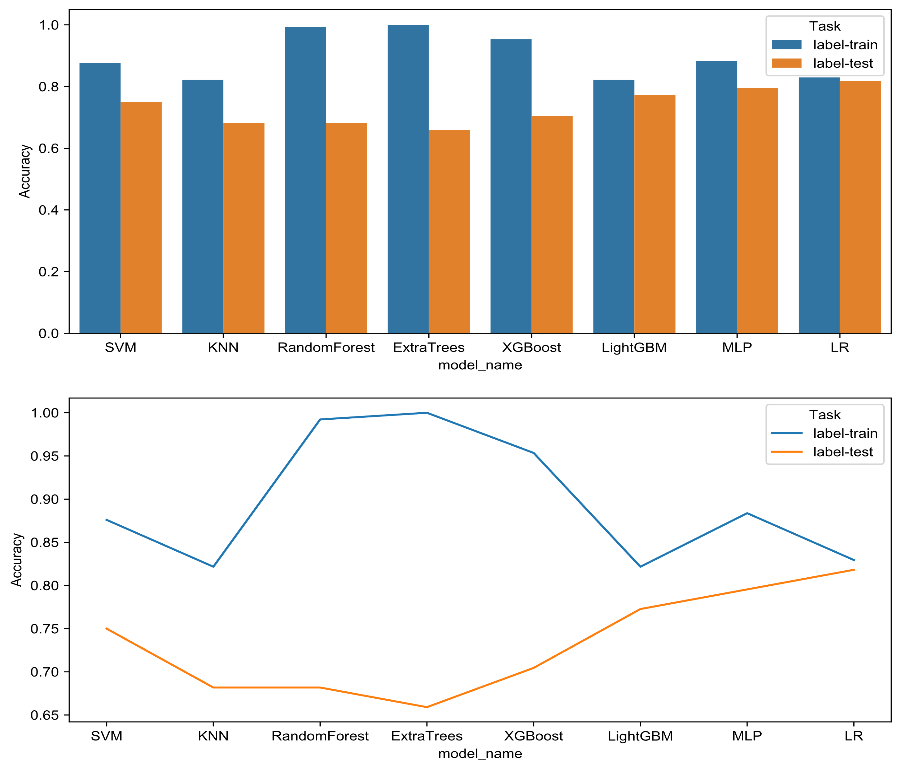


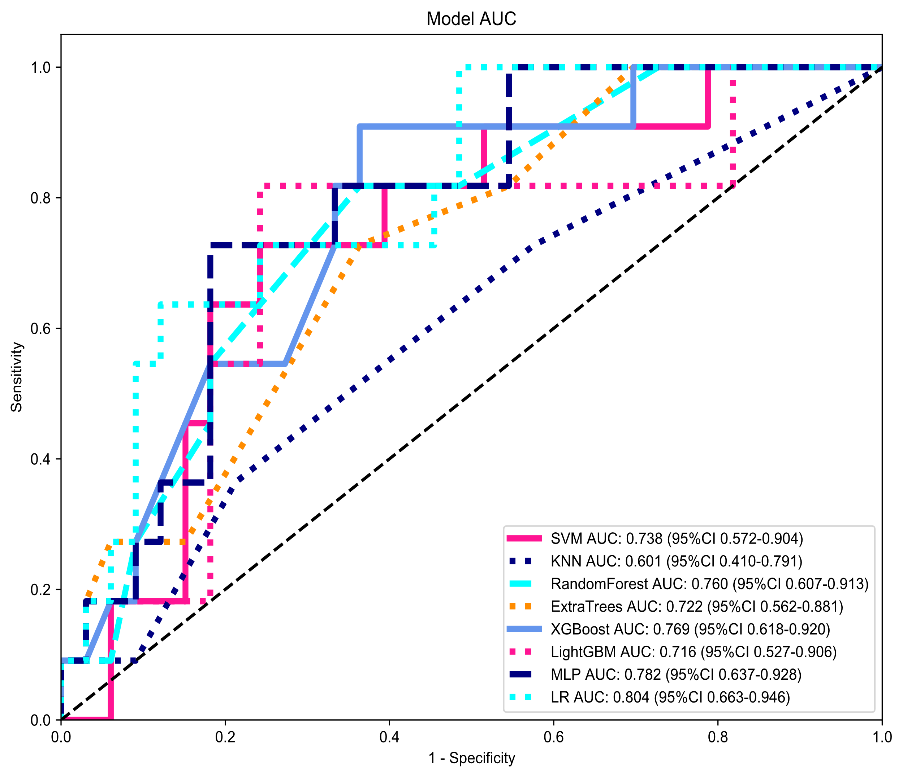
Figure 1 Accuracy curve analysis of different machine learning models based on radiomics features

Figure 2 Performance of machine learning-based radiomics models in the test set ROC, receiver operating characteristic.

**Signature**

The mean and variance (STD) of each column of features are obtained after the deep learning feature has been compressed using PCA and standardized by the Z-score method. A standard normal distribution is created by subtracting the mean from each feature column, dividing by the variance, and squaring the result. To filter out features whose coefficients are not 0, select and minimize the dimension of fusion features, and identify the ideal subset of fusion features, we apply the least absolute shrinkage and selection operator (lasso).


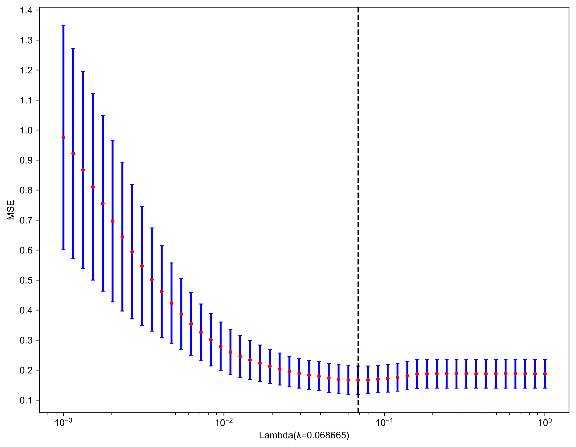
**Deep transfer learning model construction and evaluation**


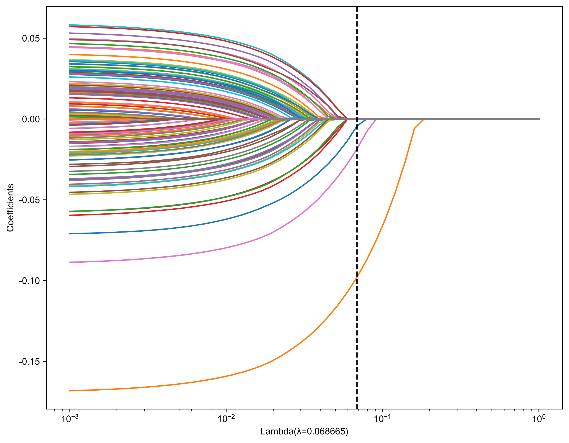


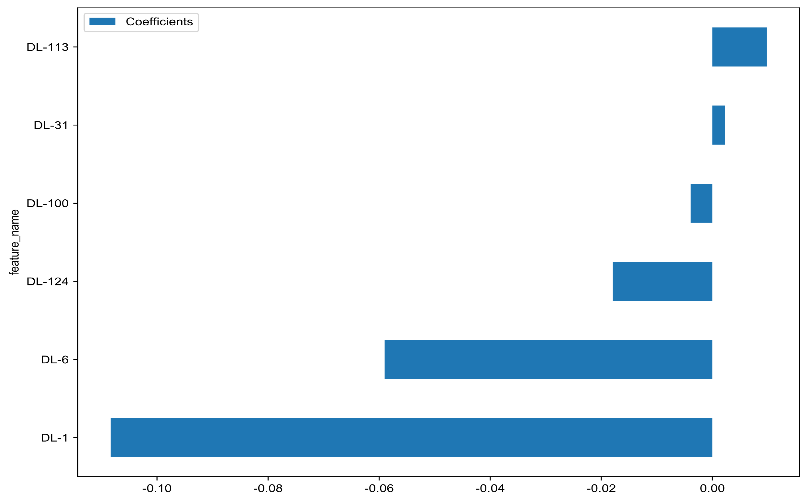
Fig3. a MSE of cross validation. b Based on the optimal λ value of 0.0449 with log(λ) = 0.68665features were selected.

Fig 4. Histogram of Rad-score based on selected Deep transfer learning.


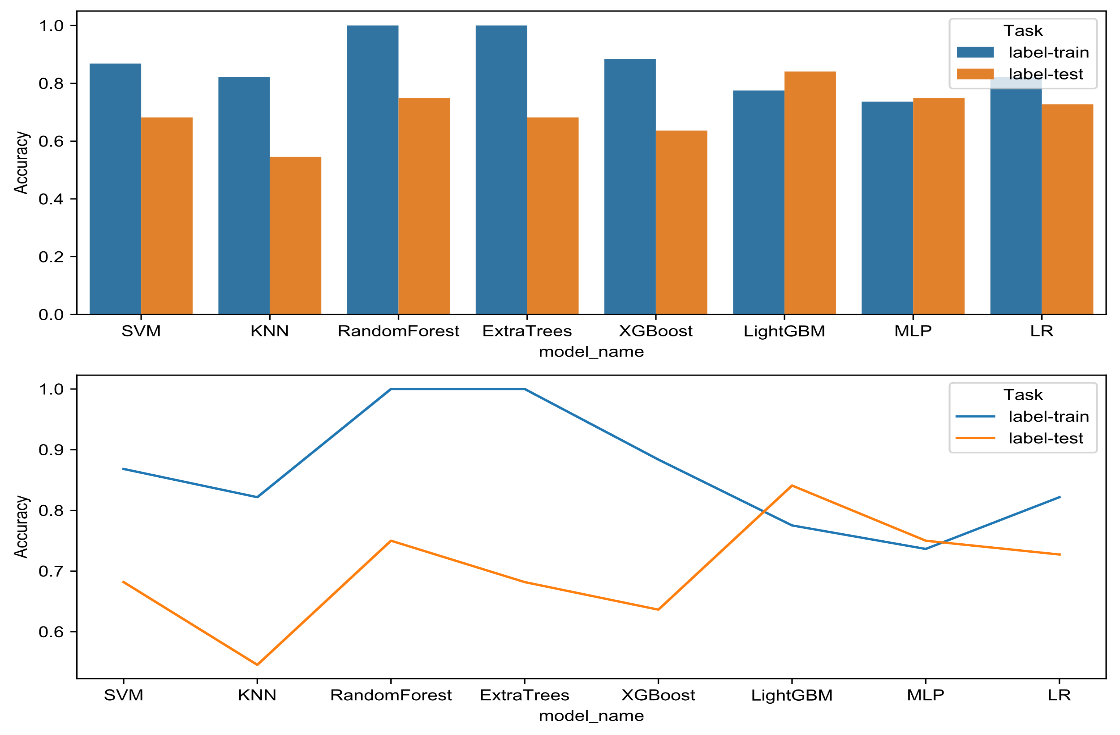


Figure 5 Accuracy curve analysis of different machine learning models based on deep migration learning features


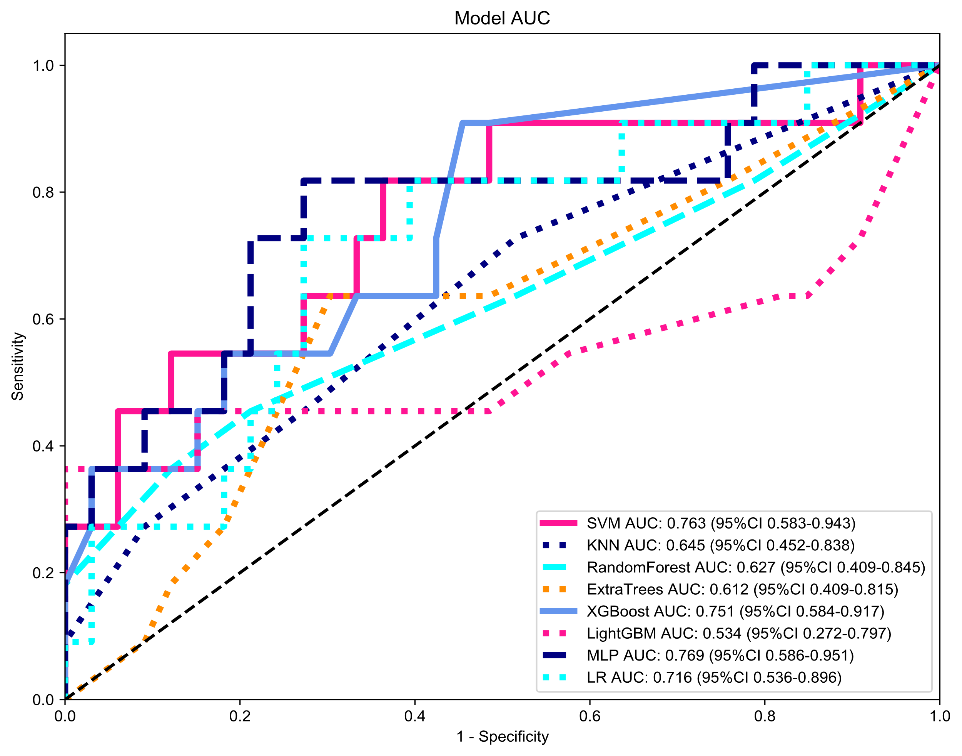


Figure 6 Performance of machine learning-based deep transfer learning models in the test set ROC, receiver operating characteristic.

**Radiomic feature extraction**

imageType:

Original: {}

LoG:

sigma: [1.0, 2.0, 3.0]

Wavelet: {}

LBP3D: {}

Exponential: {}

Square: {}

SquareRoot: {}

Logarithm: {}

Gradient: {}

featureClass:

shape:

firstorder:

glcm: # Disable SumAverage by specifying all other GLCM features available

- 'Autocorrelation'

- 'JointAverage'

- 'ClusterProminence'

- 'ClusterShade'

- 'ClusterTendency'

- 'Contrast'

- 'Correlation'

- 'DifferenceAverage'

- 'DifferenceEntropy'

- 'DifferenceVariance'

- 'JointEnergy'

- 'JointEntropy'

- 'Imc1'

- 'Imc2'

- 'Idm'

- 'Idmn'

- 'Id'

- 'Idn'

- 'InverseVariance'

- 'MaximumProbability'

- 'SumEntropy'

- 'SumSquares'

glrlm:

glszm:

gldm:

setting:

normalize: true

normalizeScale: 1000

correctMask: true

binWidth: 5

voxelArrayShift: 1000
